# Crystal structure of poly[(2,2′-bi­pyridine-κ^2^ *N*,*N*′)tetra­kis­(μ-cyanido-κ^2^ *N*:*C*)dinickel(II)]

**DOI:** 10.1107/S2056989015009706

**Published:** 2015-05-28

**Authors:** Minghui Zuo, Haiyu Wang, Jie Xu, Lingling Zhu, Shuxin Cui

**Affiliations:** a College of Chemistry and Chemical Engineering, Mu Danjiang Normal University, Mu Danjiang 157012, People’s Republic of China

**Keywords:** crystal structure, cyanide ligands, nickel, 2,2′-bi­pyridine, coordination polymer

## Abstract

The polymeric title complex, [Ni_2_(CN)_4_(C_10_H_8_N_2_)]_*n*_, was obtained serendipitously under hydro­thermal conditions. The asymmetric unit consists of one half of an [Ni(CN)_4_]^2−^ anion with the Ni^2+^ cation situated on an inversion centre, and one half of an [Ni(2,2′-bpy)]^2+^ cation (2,2′-bpy is 2,2′-bi­pyridine), with the second Ni^2+^ cation situated on a twofold rotation axis. The two Ni^2+^ cations exhibit different coordination spheres. Whereas the coordination of the metal in the anion is that of a slightly distorted square defined by four C-bound cyanide ligands, the coordination in the cation is that of a distorted octa­hedron defined by four N-bound cyanide ligands and two N atoms from the chelating 2,2′-bpy ligand. The two different Ni^2+^ cations are alternately bridged by the cyanide ligands, resulting in a two-dimensional structure extending parallel to (010). Within the sheets, π–π inter­actions between pyridine rings of neighbouring 2,2′-bpy ligands, with a centroid-to-centroid distance of 3.687 (3) Å, are present. The crystal packing is dominated by van der Waals forces. A weak C—H⋯N inter­action between adjacent sheets is also observed.

## Abstract 2

The binuclear coordination polymer consists of two nickel cations with different coordination environments. One has a square-planar environment whereas the other has an octa­hedral environment. Cyanide ligands bridge the cations into a polymeric layer structure.

## Chemical context   

Coordination metal complexes have been the subject of intensive investigation not only due to their potential application to material science as catalytic, conductive, luminescent, magnetic, porous, chiral or non-linear optical materials (Janiak *et al.*, 2003 ▸), but also because of their intriguing structural diversity (Kong *et al.*, 2008 ▸). The assembly of functional mol­ecular building blocks into crystalline polymeric materials through coordination bonds or other weak inter­actions has many advantages over traditional stepwise syntheses and was demonstrated to be an effective approach to fabricating new materials (Kopotkov *et al.*, 2014 ▸). Using this approach, numerous materials with inter­esting structures and properties have been prepared through the reactions of cyanidometallate building blocks (Cui *et al.*, 2011 ▸; Zhang & Lachgar, 2015 ▸). These compounds show novel functionalities due to strong inter­actions mediated by the linear cyanide bridges. The probably oldest and most inter­esting example is the Prussian blue framework, Fe_4_[Fe(CN)_6_]_3_·14H_2_O, and its analogues derived from the assembly of hexa­cyanidometalate anions [*M*(CN)_6_]_*n*_ and transition-metal ions (Li *et al.*, 2008 ▸). For instance, cyanide-bridged bimetallic assemblies were obtained from K_3_[Fe(CN)_6_] as a source of cyanidometalate anions, metal cations, and aromatic nitro­gen-containing ligands. These compounds show inter­esting magnetic and other properties that can be affected through the careful choice of the building-block components (Shen *et al.*, 2014 ▸).
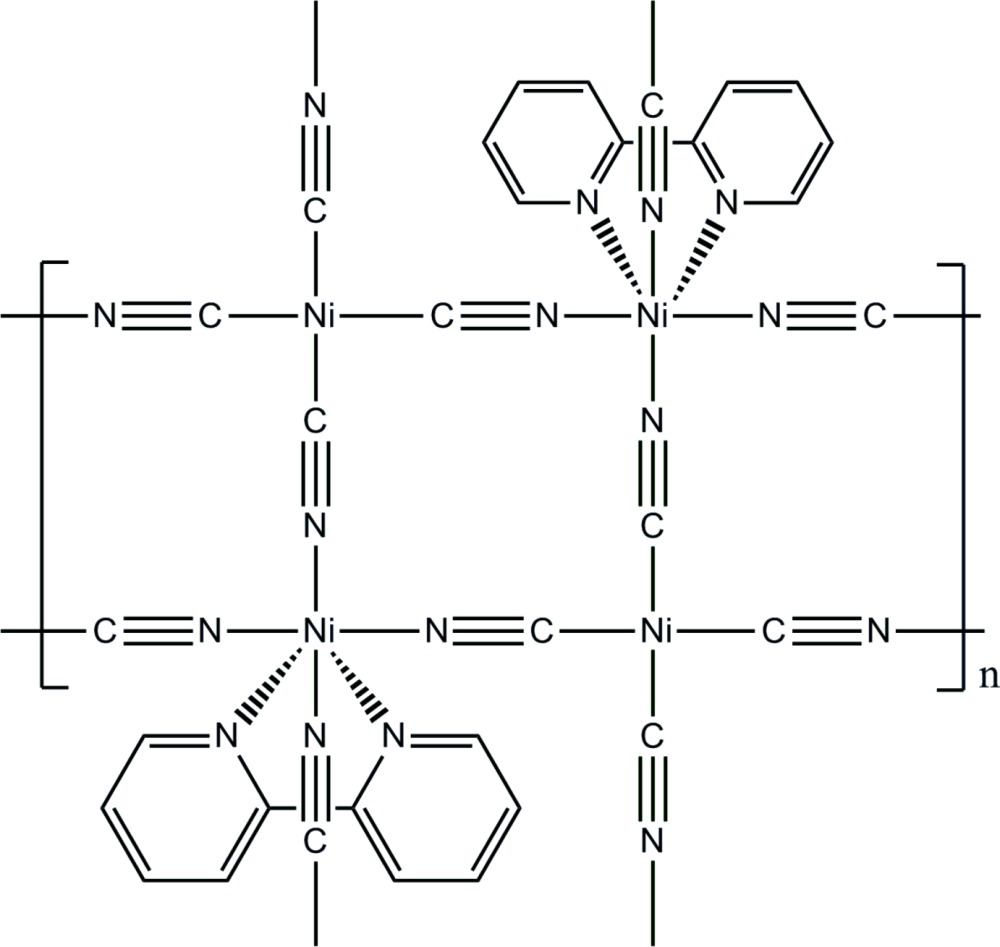


Our own efforts are focused to assemble metallic complexes and the achievement of tuning their properties by crystal engineering of the terminal/bridging ligands. However, the hydro­thermal reaction of Ni(acetate)_2_, 2,2′-bi­pyridine and K_3_[Fe(CN)_6_] did not yield the expected bimetallic system analogous to coordinated iron ions which were reported in literature (Colacio *et al.*, 2003 ▸), but to the serendipitous formation of the polymeric complex (I), [Ni_2_(CN)_4_(C_10_H_8_N_2_)]_*n*_, the crystal structure of which is reported here.

## Structural commentary   

**Figure 1:**
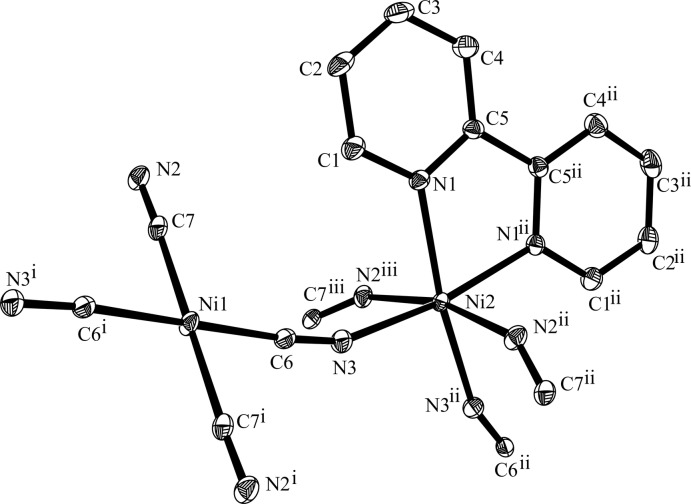
The principal building units of complex (I), showing the atom-numbering scheme. Displacement ellipsoids are drawn at the 50% probability level and H atoms have been omitted for clarity. For symmetry codes, see text.

**Figure 2:**
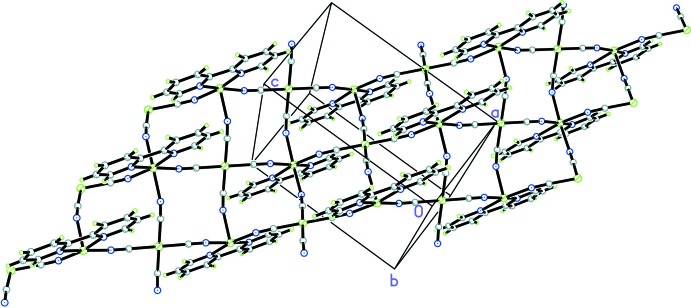
A view of the polymeric sheet of complex (I). Ni atoms are represented by hatched green spheres, C atoms are grey, N atoms blue and H atoms green.

The asymmetric unit of the structure of (I) contains formally one half of an [Ni(CN)_4_]^2−^ (Ni1) anion, and one half of an [Ni(2,2′-bpy)]^2+^ (Ni2) cation (2,2′-bpy is 2,2′-bi­pyridine). The anion is completed by inversion symmetry, whereas the cation is completed by a twofold rotation axis (Fig. 1 ▸). The Ni1 atom shows a slightly distorted square-planar geometry through coordination by four C atoms (C6 and C6^i^, C7 and C7^i^) [symmetry code: (i)*x* + 2, −*y*, −*z* + 1] from four cyanide groups, bridging Ni1 to four adjacent Ni2 atoms. The latter exhibits an overall distorted octa­hedral environment, being defined by four N atoms (N3, N3^ii^, N2^ii^, N2^iii^) [symmetry codes: (ii) −*x* + 1, *y*, −*z* + 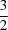; (iii) *x* − 1, *y*, *z*] from four [Ni(CN)_4_]^2−^ groups, and two N atoms (N1 and N1^ii^) of one 2,2′-bpy ligand. The corresponding N1—Ni2—N1 bite angle is 77.32 (13)°. Relevant bond lengths involving the two metal cations are compiled in Table 1 ▸. As depicted in Fig. 2 ▸, the cyanide groups bridge nickel cations, forming undulating sheets of composition [Ni_2_(CN)_4_(2,2′-bpy)_2_] parallel to (010), constituted by alternation of Ni1 and Ni2 ions.

## Supra­molecular features   

Within a sheet, π–π inter­actions between pyridine rings with a centroid-to-centroid distance of 3.687 (3) Å are present. The adhesion of the sheets in the crystal packing is dominated by van der Waals forces. However, a weak non-classical C—H⋯N inter­action (Table 2 ▸) between neighbouring sheets may participate in the stabilization of the crystal packing.

## Synthesis and crystallization   

Ni(acetate)_2_ (0.159 g, 0.64 mmol), 2,2′-bi­pyridine (0.047 g, 0.3 mmol) and K_3_[Fe(CN)_6_] (0.21 g, 0.64 mmol) dissolved in aqueous solution of 1*M* NaCl (8 ml) were added to a 15 ml Teflon-lined autoclave and heated at 433 K for 3 d. The autoclave was then cooled to room temperature. Green block-shaped crystals of (I) deposited on the wall of the container and were collected and air-dried.

## Refinement   

Crystal data, data collection and structure refinement details are summarized in Table 3 ▸. Hydrogen atoms bound to carbon were found in a difference map and were refined with *U*_iso_(H) = 1.2*U*_eq_(C).

## Supplementary Material

Crystal structure: contains datablock(s) I, global. DOI: 10.1107/S2056989015009706/wm5162sup1.cif

Structure factors: contains datablock(s) I. DOI: 10.1107/S2056989015009706/wm5162Isup2.hkl

CCDC reference: 1058383

Additional supporting information:  crystallographic information; 3D view; checkCIF report

## Figures and Tables

**Table 1:** Selected bond lengths ()

Ni1C6	1.863(3)	Ni2N1	2.102(2)
Ni1C7	1.871(3)	Ni2N2^i^	2.116(2)
Ni2N3	2.071(2)		

Symmetry code: (i) 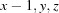.

**Table 2:** Hydrogen-bond geometry (, )

*D*H*A*	*D*H	H*A*	*D* *A*	*D*H*A*
C1H1N3	0.96(3)	2.54(3)	3.129(3)	120(2)

**Table 3:** Experimental details

Crystal data	
Chemical formula	[Ni_2_(CN)_4_(C_10_H_8_N_2_)]
*M*_r_	377.68
Crystal system, space group	Monoclinic, *C*2/*c*
Temperature (K)	293
*a*, *b*, *c* ()	6.519(5), 16.698(5), 12.019(5)
()	90.852(5)
*V* (^3^)	1308.2(12)
*Z*	4
Radiation type	Mo *K*
(mm^1^)	2.88
Crystal size (mm)	0.40 0.10 0.06

Data collection	
Diffractometer	Siemens SMART CCD
No. of measured, independent and observed [*I* > 2(*I*)] reflections	3858, 1156, 1039
*R*_int_	0.032
(sin /)_max_ (^1^)	0.594

Refinement	
*R*[*F* ^2^ > 2(*F* ^2^)], *wR*(*F* ^2^), *S*	0.028, 0.074, 1.10
No. of reflections	1156
No. of parameters	118
H-atom treatment	H atoms treated by a mixture of independent and constrained refinement
_max_, _min_ (e ^3^)	0.71, 0.40

Computer programs: *SMART* and *SAINT* (Bruker, 2007 ▸), *SHELXS97*, *SHELXL97*and *XP* in *SHELXTL* (Sheldrick, 2008 ▸).

## supplementary crystallographic information

### Crystal data

**Table:**

[Ni_2_(CN)_4_(C_10_H_8_N_2_)]	*F*(000) = 760
*M**_r_* = 377.68	*D*_x_ = 1.918 Mg m^−^^3^
Monoclinic, *C*2/*c*	Mo *K*α radiation, λ = 0.71069 Å
Hall symbol: -C 2yc	Cell parameters from 3858 reflections
*a* = 6.519 (5) Å	θ = 1.0–25.0°
*b* = 16.698 (5) Å	µ = 2.88 mm^−^^1^
*c* = 12.019 (5) Å	*T* = 293 K
β = 90.852 (5)°	Block, green
*V* = 1308.2 (12) Å^3^	0.40 × 0.10 × 0.06 mm
*Z* = 4	

### Data collection

**Table:**

Siemens SMART CCD diffractometer	1039 reflections with *I* > 2σ(*I*)
Radiation source: fine-focus sealed tube	*R*_int_ = 0.032
Graphite monochromator	θ_max_ = 25.0°, θ_min_ = 3.4°
Detector resolution: 9 pixels mm^-1^	*h* = −7→7
ω scans	*k* = −19→16
3858 measured reflections	*l* = −14→10
1156 independent reflections	

### Refinement

**Table:**

Refinement on *F*^2^	Primary atom site location: structure-invariant direct methods
Least-squares matrix: full	Secondary atom site location: difference Fourier map
*R*[*F*^2^ > 2σ(*F*^2^)] = 0.028	Hydrogen site location: inferred from neighbouring sites
*wR*(*F*^2^) = 0.074	H atoms treated by a mixture of independent and constrained refinement
*S* = 1.10	*w* = 1/[σ^2^(*F*_o_^2^) + (0.0374*P*)^2^ + 0.9543*P*] where *P* = (*F*_o_^2^ + 2*F*_c_^2^)/3
1156 reflections	(Δ/σ)_max_ < 0.001
118 parameters	Δρ_max_ = 0.71 e Å^−^^3^
0 restraints	Δρ_min_ = −0.40 e Å^−^^3^

### Special details

**Table:**

Geometry. All e.s.d.'s (except the e.s.d. in the dihedral angle between two l.s. planes) are estimated using the full covariance matrix. The cell e.s.d.'s are taken into account individually in the estimation of e.s.d.'s in distances, angles and torsion angles; correlations between e.s.d.'s in cell parameters are only used when they are defined by crystal symmetry. An approximate (isotropic) treatment of cell e.s.d.'s is used for estimating e.s.d.'s involving l.s. planes.
Refinement. Refinement of *F*^2^ against ALL reflections. The weighted *R*-factor *wR* and goodness of fit *S* are based on *F*^2^, conventional *R*-factors *R* are based on *F*, with *F* set to zero for negative *F*^2^. The threshold expression of *F*^2^ > σ(*F*^2^) is used only for calculating *R*-factors(gt) *etc*. and is not relevant to the choice of reflections for refinement. *R*-factors based on *F*^2^ are statistically about twice as large as those based on *F*, and *R*- factors based on ALL data will be even larger.

### Fractional atomic coordinates and isotropic or equivalent isotropic displacement parameters (Å^2^)

**Table:**

	*x*	*y*	*z*	*U*_iso_*/*U*_eq_	
Ni1	1.0000	0.0000	0.5000	0.01983 (19)	
Ni2	0.5000	0.12867 (3)	0.7500	0.01682 (18)	
C6	0.8027 (4)	0.02639 (16)	0.6043 (2)	0.0207 (6)	
C1	0.8426 (4)	0.22353 (19)	0.6347 (3)	0.0275 (7)	
N3	0.6828 (3)	0.04836 (14)	0.6654 (2)	0.0242 (5)	
N1	0.6675 (3)	0.22697 (13)	0.6906 (2)	0.0198 (5)	
C5	0.5950 (4)	0.29952 (16)	0.7170 (2)	0.0207 (6)	
C7	1.1817 (4)	0.07283 (17)	0.5669 (2)	0.0220 (6)	
C4	0.6937 (5)	0.36889 (18)	0.6855 (3)	0.0305 (7)	
N2	1.2931 (3)	0.11329 (14)	0.6143 (2)	0.0240 (5)	
C3	0.8723 (5)	0.3640 (2)	0.6262 (3)	0.0343 (8)	
C2	0.9471 (5)	0.29004 (19)	0.6008 (3)	0.0317 (7)	
H2	1.062 (5)	0.284 (2)	0.562 (3)	0.043 (10)*	
H4	0.636 (4)	0.4223 (19)	0.711 (3)	0.031 (8)*	
H1	0.892 (5)	0.171 (2)	0.616 (3)	0.033 (9)*	
H3	0.940 (5)	0.409 (2)	0.608 (3)	0.038 (9)*	

### Atomic displacement parameters (Å^2^)

**Table:**

	*U*^11^	*U*^22^	*U*^33^	*U*^12^	*U*^13^	*U*^23^
Ni1	0.0146 (3)	0.0249 (3)	0.0202 (3)	−0.00164 (17)	0.0062 (2)	−0.0069 (2)
Ni2	0.0146 (3)	0.0183 (3)	0.0177 (3)	0.000	0.0058 (2)	0.000
C6	0.0184 (13)	0.0189 (14)	0.0248 (16)	−0.0028 (11)	0.0033 (12)	−0.0031 (12)
C1	0.0244 (15)	0.0288 (17)	0.0297 (18)	−0.0016 (12)	0.0091 (13)	−0.0005 (13)
N3	0.0212 (12)	0.0240 (13)	0.0275 (15)	0.0002 (9)	0.0066 (11)	−0.0042 (11)
N1	0.0186 (11)	0.0217 (12)	0.0192 (13)	−0.0020 (9)	0.0026 (9)	0.0021 (10)
C5	0.0217 (14)	0.0197 (15)	0.0208 (16)	−0.0016 (10)	−0.0013 (12)	0.0022 (11)
C7	0.0176 (13)	0.0254 (15)	0.0233 (16)	0.0018 (11)	0.0084 (12)	−0.0024 (12)
C4	0.0314 (17)	0.0264 (17)	0.034 (2)	−0.0035 (12)	0.0005 (14)	0.0030 (13)
N2	0.0187 (12)	0.0289 (13)	0.0245 (15)	−0.0021 (10)	0.0054 (10)	−0.0033 (11)
C3	0.0331 (18)	0.0323 (19)	0.037 (2)	−0.0125 (14)	0.0004 (15)	0.0098 (15)
C2	0.0241 (16)	0.0390 (19)	0.0323 (19)	−0.0083 (13)	0.0084 (14)	0.0054 (15)

### Geometric parameters (Å, º)

**Table:**

Ni1—C6^i^	1.863 (3)	C1—C2	1.368 (4)
Ni1—C6	1.863 (3)	C1—H1	0.95 (3)
Ni1—C7^i^	1.871 (3)	N1—C5	1.340 (3)
Ni1—C7	1.871 (3)	C5—C4	1.381 (4)
Ni2—N3^ii^	2.071 (2)	C5—C5^ii^	1.480 (5)
Ni2—N3	2.071 (2)	C7—N2	1.139 (4)
Ni2—N1	2.102 (2)	C4—C3	1.377 (5)
Ni2—N1^ii^	2.102 (2)	C4—H4	1.02 (3)
Ni2—N2^iii^	2.116 (2)	N2—Ni2^v^	2.116 (2)
Ni2—N2^iv^	2.116 (2)	C3—C2	1.364 (5)
C6—N3	1.140 (4)	C3—H3	0.90 (4)
C1—N1	1.335 (4)	C2—H2	0.89 (4)
			
C6^i^—Ni1—C6	180.0	N1—C1—C2	123.3 (3)
C6^i^—Ni1—C7^i^	89.76 (12)	N1—C1—H1	117 (2)
C6—Ni1—C7^i^	90.24 (12)	C2—C1—H1	120 (2)
C6^i^—Ni1—C7	90.24 (12)	C6—N3—Ni2	158.3 (2)
C6—Ni1—C7	89.76 (12)	C1—N1—C5	117.7 (2)
C7^i^—Ni1—C7	180.00 (13)	C1—N1—Ni2	126.15 (19)
N3^ii^—Ni2—N3	99.29 (14)	C5—N1—Ni2	116.02 (18)
N3^ii^—Ni2—N1	167.94 (10)	N1—C5—C4	121.7 (3)
N3—Ni2—N1	91.91 (10)	N1—C5—C5^ii^	115.32 (15)
N3^ii^—Ni2—N1^ii^	91.91 (10)	C4—C5—C5^ii^	122.95 (18)
N3—Ni2—N1^ii^	167.94 (10)	N2—C7—Ni1	174.8 (3)
N1—Ni2—N1^ii^	77.32 (13)	C3—C4—C5	119.6 (3)
N3^ii^—Ni2—N2^iii^	86.28 (10)	C3—C4—H4	122.1 (18)
N3—Ni2—N2^iii^	84.71 (10)	C5—C4—H4	118.2 (18)
N1—Ni2—N2^iii^	99.29 (9)	C7—N2—Ni2^v^	148.4 (2)
N1^ii^—Ni2—N2^iii^	91.61 (9)	C2—C3—C4	118.5 (3)
N3^ii^—Ni2—N2^iv^	84.71 (10)	C2—C3—H3	122 (2)
N3—Ni2—N2^iv^	86.28 (10)	C4—C3—H3	120 (2)
N1—Ni2—N2^iv^	91.61 (9)	C3—C2—C1	119.1 (3)
N1^ii^—Ni2—N2^iv^	99.29 (9)	C3—C2—H2	122 (2)
N2^iii^—Ni2—N2^iv^	166.06 (13)	C1—C2—H2	119 (2)
N3—C6—Ni1	174.8 (2)		
			
N3^ii^—Ni2—N3—C6	173.2 (7)	N3—Ni2—N1—C5	174.8 (2)
N1—Ni2—N3—C6	−11.3 (6)	N1^ii^—Ni2—N1—C5	0.33 (14)
N1^ii^—Ni2—N3—C6	15.2 (9)	N2^iii^—Ni2—N1—C5	89.9 (2)
N2^iii^—Ni2—N3—C6	87.8 (6)	N2^iv^—Ni2—N1—C5	−98.8 (2)
N2^iv^—Ni2—N3—C6	−102.8 (6)	C1—N1—C5—C4	1.7 (4)
C2—C1—N1—C5	−1.9 (5)	Ni2—N1—C5—C4	178.3 (2)
C2—C1—N1—Ni2	−178.2 (2)	C1—N1—C5—C5^ii^	−177.6 (3)
N3^ii^—Ni2—N1—C1	149.5 (4)	Ni2—N1—C5—C5^ii^	−0.9 (4)
N3—Ni2—N1—C1	−8.8 (2)	N1—C5—C4—C3	−0.6 (5)
N1^ii^—Ni2—N1—C1	176.7 (3)	C5^ii^—C5—C4—C3	178.6 (3)
N2^iii^—Ni2—N1—C1	−93.7 (2)	C5—C4—C3—C2	−0.3 (5)
N2^iv^—Ni2—N1—C1	77.5 (2)	C4—C3—C2—C1	0.1 (5)
N3^ii^—Ni2—N1—C5	−26.9 (5)	N1—C1—C2—C3	1.0 (5)

Symmetry codes: (i) −*x*+2, −*y*, −*z*+1; (ii) −*x*+1, *y*, −*z*+3/2; (iii) *x*−1, *y*, *z*; (iv) −*x*+2, *y*, −*z*+3/2; (v) *x*+1, *y*, *z*.

### Hydrogen-bond geometry (Å, º)

**Table:**

*D*—H···*A*	*D*—H	H···*A*	*D*···*A*	*D*—H···*A*
C1—H1···N3	0.96 (3)	2.54 (3)	3.129 (3)	120 (2)
